# Umbrella Reviews: Concepts, Methodological Frameworks, and Step‐by‐Step Implementation

**DOI:** 10.1111/jebm.70092

**Published:** 2025-12-16

**Authors:** Chang Liu, Dayang Zhou, Wenjing Xu, Hai Pan, Xueqin Wang, Jibang Peng, Xiang Ji, Jian Huang, Zhu Zhu

**Affiliations:** ^1^ Day Surgery Center First Affiliated Hospital of Kunming Medical University Kunming Yunnan China; ^2^ Department of Surgical Oncology First Affiliated Hospital of Kunming Medical University Kunming Yunnan China

**Keywords:** evidence‐based medicine, meta‐analysis, systematic review, umbrella review

## Abstract

The exponential growth of secondary literature has created an imperative for researchers to identify credible and methodologically sound studies within an increasingly complex information landscape. In light of the growing emphasis on evidence‐based medicine in both domestic and international contexts, umbrella reviews (URs) have emerged as a critical methodological approach in biomedical research. As an advanced form of tertiary evidence synthesis, URs systematically integrate findings from multiple systematic reviews and meta‐analyses, thereby providing a comprehensive evidence base for specific research questions or related fields. This methodological framework enhances the quality of evidence through critically evaluating the validity and reliability of conclusions drawn from secondary or primary studies. The present overview systematically examines the conceptual framework, distinctive characteristics, development process, and methodological quality assessment of URs, with the objective of establishing a theoretical foundation and practical reference for future research in this domain.

## 1 Introduction

Etiological prevention, alongside disease diagnosis and treatment, constitutes a fundamental focus in medical and clinical healthcare research. To address these critical aspects, numerous investigators have utilized evidence‐based medicine (EBM) methodologies to systematically examine exposure‐outcome relationships through epidemiological investigations. These studies have been conducted employing varied research strategies and orientations, thereby generating medical evidence of differential quality and yielding multifaceted conclusions from diverse analytical perspectives.

Systematic reviews (SRs) and meta‐analyses (MAs) represent the highest tier of secondary research evidence and serve as a fundamental pillar of EBM [1, 2]. Over the past two decades, SRs and MAs (collectively referred to as SRMAs) have emerged as the predominant methodology for synthesizing evidence across diverse domains of clinical and epidemiological research, including interventions, diagnostic studies, etiological investigations, and population health metrics. However, the exponential growth in the publication of secondary studies has introduced significant challenges. The analysis and curation of aggregated data from multiple SRMAs not only impose substantial time and resource burdens on researchers but also hinder the ability of healthcare decision‐makers to promptly identify and implement the most robust evidence‐based practices [3, 4]. According to partial data, the PubMed database has indexed over 510,000 SRMAs (as of October 30, 2025, using the search terms “(meta analysis [Title/Abstract]) OR (systematic review [Title/Abstract])”). Within this expansive and heterogeneous body of evidence, the task of discerning genuinely high‐quality and reliable findings has become increasingly complex and critical.

In addressing this critical challenge, umbrella reviews (URs), defined as systematic syntheses of existing SRs, were formally introduced in the late 1990s [5, 6, 7] and subsequently refined through the development of key methodological frameworks. URs have been extensively utilized to synthesize evidence across multiple SRs, particularly in specialized domains such as cancer biomarker research, where they have facilitated the consolidation of immunohistochemistry data, thereby underscoring their significance in EBM [8]. Methodological advancements in URs include the establishment of quality assessment tools by Lun Li et al. [9], the development of the Joanna Briggs Institute (JBI) framework for conducting and reporting URs by Edoardo Aromataris et al. [10], and the formulation of “10 simple rules” for UR design by Paolo Fusar‐Poli et al. [11]. Nevertheless, several methodological limitations persist: insufficient guidance on integrating heterogeneous evidence types; a lack of consensus on addressing overlapping primary studies across SRMAs, prevalent issue in fields such as cancer biomarkers; and inconsistent application of the Grade of Recommendations Assessment, Development and Evaluation (GRADE) framework for cross‐review evidence assessment, among others. Meanwhile, various disciplines, both domestically and internationally, are vigorously engaged in the development of high‐quality SRMAs. According to preliminary data from PubMed, the number of published URs articles has exceeded 3000 and continues to grow annually (as of October 30, 2025, using the search term “umbrella review [Title/Abstract]”). Therefore, clinical practitioners must systematically master and proficiently apply the methodological framework of URs to efficiently identify the highest level of evidence from a vast array of evidence‐based resources and make scientifically grounded, personalized diagnostic and treatment decisions.

Consequently, this study presents an SR of URs, with a particular emphasis on their methodological frameworks, procedure development, and analytical approaches, aiming to provide a significant theoretical foundation and practical reference for advancing high‐quality evidence synthesis and establishing robust evidence‐based practices. It is worth mentioning that the overall structural framework of this study refers to the UR methodology proposed by Belbasis et al., especially in the application of the PICOS (Population, Intervention, Comparison, Outcome, and Study Design) framework for defining research problem and the use of certain data processing tools, both of which reference their standardized process [12].

## 2 Definition and Core Concepts

As a novel approach in EBM analysis, the development of URs is closely intertwined with the evolution of SRs and its overview, collectively highlighting its increasing importance in integrating medical evidence [13].

The origins of SRs can be traced back to the emergence of EBM methodology in the 1970s and 1980s [14]. Its primary objective is to provide high‐quality evidence to support clinical decision‐making and health policy formulation through systematic methods of comprehensive literature search, screening, quality assessment, and data synthesis for specific clinical issues [15, 16]. Initial SRs focused on well‐defined, narrow research questions and often emphasized the synthesis of “stronger” types of evidence, such as randomized controlled trials (RCTs) [14]. However, with the deepening of research and the increasing complexity of interventions, this overly narrow approach may not fully capture the entire scope of complex issues and interventions. With the rapid growth in the number of SRs, concerns about their methodological quality, reliability, and applicability have emerged [17, 18]. This has prompted the need for “re‐evaluation” of SRs, involving re‐examination and critical assessment of published SRs. This re‐evaluation process has further revealed potential heterogeneity, risk of bias, and inconsistencies in results among existing SRs [19, 20]. These challenges have driven researchers to seek higher level evidence synthesis methods to address the limitations and information redundancy inherent in SRs themselves [21, 22]. In this context, the URs have emerged.

URs, also known as SRs of reviews, SRs of MAs, umbrella overviews, and comprehensive overviews, represent a methodological approach designed to systematically organize and prioritize diverse evaluation metrics within a structured framework [21, 22, 23]. This process involves the assigning of weighted values to individual indicators based on their relative significance, thereby establishing a hierarchical evaluation system analogous to an umbrella structure [24]. Through this integrative framework, each discrete evaluation metric contributes to a comprehensive assessment of the research subject, which not only achieves a contextualized understanding of the research object but also effectively mitigates the inherent limitations of traditional unified evaluation paradigms.

**FIGURE 1:**
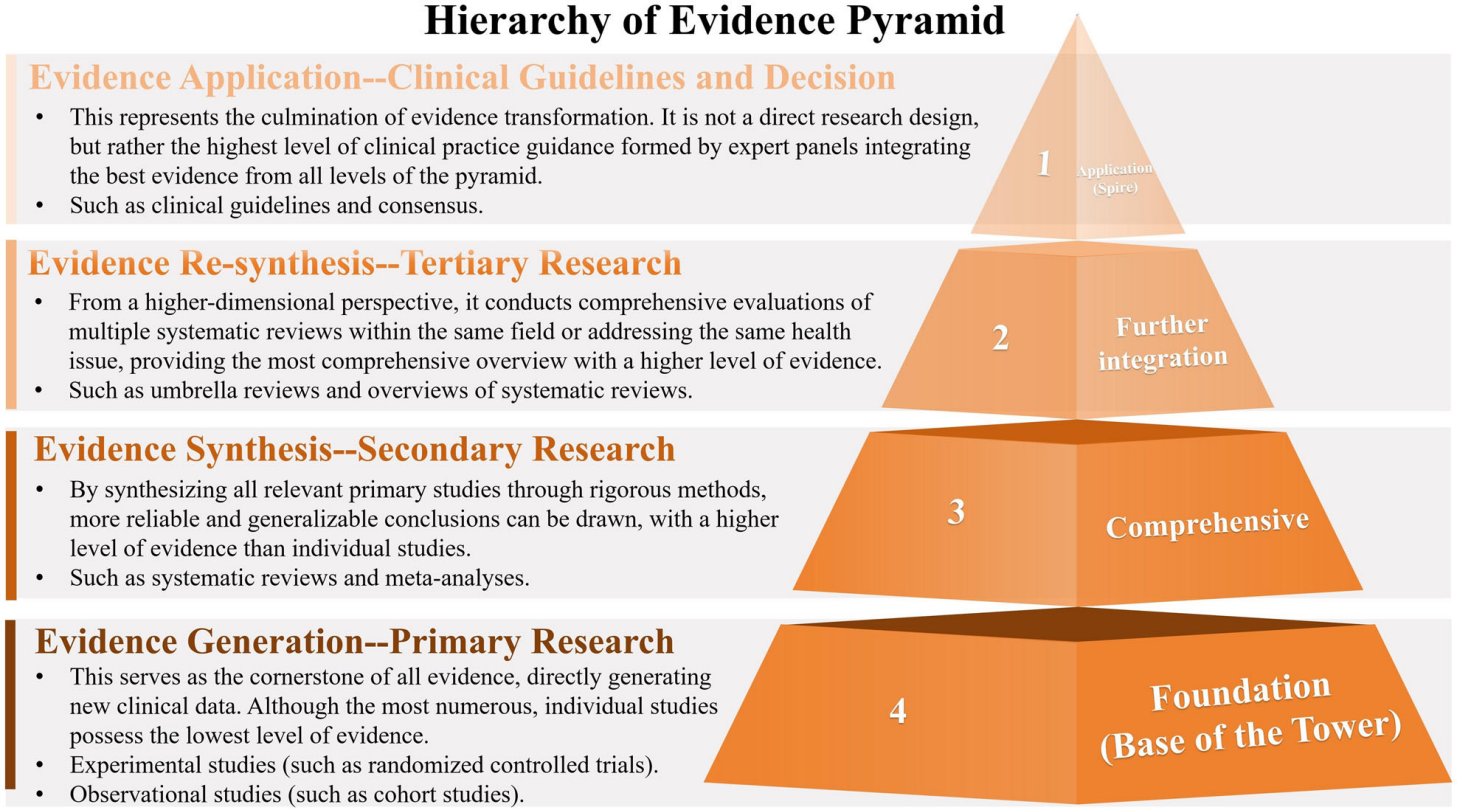
Hierarchical relationships in evidence synthesis in evidence‐based medicine.

URs constitute a methodological progression in research synthesis, building upon and refining traditional evaluation frameworks to enhance the objectivity and comprehensiveness of assessment outcomes (Figure 1) [25, 26]. Functioning through a systematic approach analogous to SRMAs, URs synthesize and reanalyze existing data from multiple SRMAs to generate a more comprehensive and authoritative evidence base [10, 27]. This methodological framework is particularly suited for addressing specific topics within a research field, thereby facilitating evidence‐based conclusions. Notably, the application of URs is warranted only when there is a substantial number of SRMAs on a particular research topic and when their findings exhibit significant heterogeneity or controversy [28].

## 3 Comparing With Other Reviews

URs and SRMAs occupy different levels in the process of evidence synthesis, with their core differences manifesting in research objectives and methodologies. SRMAs aim to address a specific research question (e.g., the impact of an intervention on a particular outcome) by directly synthesizing raw study data for quantitative or qualitative analysis, providing specific effect estimates (e.g., risk ratios); their quality assessment focuses on examining the risk of bias in primary studies [29, 30, 31]. In contrast, URs operate at a higher level of evidence integration, as they do not directly handle primary studies but instead use published SRMAs as the basic unit, aiming to evaluate the overall evidence landscape in a broad field. Consequently, their analytical approach emphasizes comparing and summarizing the conclusions, methodological quality (e.g., using the A MeaSurement Tool to Assess SRs‐2 (AMSTAR‐2) tool), and evidence consistency of these SRs, presenting a macro overview through evidence maps and strength grading rather than recalculating effect sizes [21, 32, 33]. In summary, SRMAs serve as the cornerstone for addressing specific research questions, whereas URs are advanced tools providing a panoramic evidence view for macro‐level decision‐making, forming a complementary and progressive relationship within the evidence ecosystem.

Meanwhile, the URs differ from the overview of SRs, which also belongs to the tertiary level of research. The latter primarily focuses on organizing and summarizing existing SRs, addressing multiple SRs on the same clinical issue, thereby facilitating a quick understanding of the current research status of a particular intervention or problem [36]. In contrast, the former integrates multiple SRMAs to conduct a comprehensive assessment of a broad field, providing a panoramic evidence map of a topic to compare the strength and consistency of evidence for different interventions within that field. Table 1 illustrates the differences among these three approaches [17, 36].

**TABLE 1:** Comparative analysis of systematic reviews, overview umbrella reviews, and systematic review/meta‐analysis.

Point of distinction	Overview of systematic reviews	URs	Systematic review/meta‐analysis
Research object	Systematic review/meta‐analysis	Systematic review/meta‐analysis	Primary research
Choice of topics	Highly focused and specific	Relatively broad	Highly focused and specific
Literature included	Multiple systematic reviews/meta‐analyses addressing the same specific clinical question	Multiple systematic reviews/meta‐analyses within the same broad field, each addressing a specific question within that field.	Multiple original studies addressing the same clinical issue
Focus	Comprehensively collect and synthesize multiple systematic reviews addressing the same clinical question, aiming to provide a concise, unified summary for clinical decision‐making	Conduct a comprehensive assessment of a broad field by synthesizing multiple SRMAs to compare the strength and consistency of evidence for different interventions within that field	Answer a specific clinical question by synthesizing the effects of interventions or exposures through original research such as randomized controlled trials and cohort studies
Evaluation tools	PRISMA, AMSTAR, and AMSTAR‐2	PRISMA, GRADE, and AMSTAR‐2	Cochrane Risk of Bias Assessment Tool, MOOSE, PRISMA, etc.
Evidence‐based hierarchy	Tertiary study	Tertiary study	Secondary study

Abbreviations: AMSTAR‐2, A Measurement Tool to Assess Systematic Reviews 2; GRADE, Grading of Recommendations Assessment, Development and Evaluations; MOOSE, Meta‐analysis Of Observational Studies in Epidemiology; PRISMA, Preferred Reporting Items for Systematic Reviews and Meta‐Analyses; ROBIS, Risk Of Bias In Systematic Reviews; URs, umbrella reviews.

## 4 Application Scenarios of URs

URs represent an emerging methodology in EBM, designed to synthesize and evaluate the findings of numerous SRMAs, thereby providing a higher level of evidence synthesis for clinical practice, policy formulation, and research direction [36, 37, 38]. With the rapid proliferation of SRMAs, URs effectively address information overload and potential discrepancies among different study conclusions, offering a comprehensive and clear evidence overview for specific research topics through systematic organization and unified assessment [22, 39].

In practical applications, URs are primarily manifested in four aspects: First, they synthesize and evaluate the findings of multiple SRMAs, forming an overall evidence landscape for a particular field, and employ tools such as AMSTAR‐2 to assess the quality of included studies, ensuring the reliability of evidence [40, 41, 42]. Second, in addressing potential conflicts among different SRs, URs recalculate effect estimates and adopt a unified framework for evidence grading, effectively resolving inconsistencies and enhancing the clarity of conclusions [39, 43]. Third, URs provide high‐level evidence support for clinical decision‐making and policy formulation, assisting healthcare practitioners and policymakers in obtaining comprehensive information from complex research results to make scientific judgments [37, 44]. Fourth, they identify gaps and deficiencies in research fields, guiding future research directions, avoiding resource wastage, and promoting disciplinary development [45, 46].

In this regard, URs have demonstrated extensive application value across various professional fields [33, 47, 48, 49]. In healthcare, they are used to assess the association between temporomandibular joint disorders and ear symptoms, the intervention effects of virtual reality on cognitive impairments, and the relationship between dietary factors and gestational diabetes risk, and so forth [41, 50, 51, 52]. In education, they explore the relationship between socioeconomic status and academic performance, and so forth [53, 54]. In technology‐related areas, they involve the application of blockchain in smart grids, telemedicine evaluation, the efficacy of artificial intelligence in caries diagnosis, and so forth [55, 56, 57]. Additionally, in sports, psychology, industrial sustainable maintenance, and public health engagement, URs play a role in evidence integration [58, 59, 60]. Overall, URs, with their systematic and comprehensive evidence synthesis capabilities, demonstrate unique value in addressing complex and voluminous research evidence. They connect SRMAs from different sources into a more authoritative and macro‐evidence system, significantly promoting evidence‐based practice and scientific decision‐making across multiple disciplines [22, 36].

## 5 Advantages and Limitations of URs

URs constitute the pinnacle of evidence synthesis in EBM, integrating results from multiple SRMAs to generate comprehensive tertiary evidence. The primary strength of URs lies in their ability to flexibly set evaluation criteria and systematically integrate evidence from multiple research fields [61, 62, 63]. On the basis of existing SRMAs, they not only enhance efficiency but also strengthen the reliability of conclusions by pooling high‐quality evidence, which is of significant value in guiding clinical practice and research directions. Through clear research objectives and rigorous screening criteria, URs help researchers accurately locate relevant high‐quality evidence in a vast body of literature, thereby avoiding resource waste. They also provide a common framework and standard language for multidisciplinary collaboration, promoting team understanding and cooperation, making comprehensive results more reliable, and ultimately laying a solid empirical foundation for clinical decision‐making and policy formulation [26, 64].

However, this method also has certain limitations [63, 65]. Its conclusions are highly dependent on the methodological quality of the included SRMAs, susceptible to biases at the level of original studies and SRs, and unable to conduct in‐depth subgroup analysis based on individual participant data, sometimes requiring verification by revisiting original studies. In terms of timeliness and comprehensiveness, URs are limited by the update pace of the included SRMAs, often failing to cover the latest evidence and potentially omitting unpublished or minority language studies, leading to an incomplete evidence base. Additionally, URs still lack a unified analytical framework for addressing methodological and clinical heterogeneity among different SRMAs and have not established standardized solutions for evidence overlap issues. At the technical level, URs mainly rely on general statistical software such as R and Stata, lacking dedicated tool support, which affects the efficiency of processing massive data. Moreover, their complex processes and high requirements for methodological transparency are often inadequately reported in existing studies, urgently necessitating the development of targeted reporting standards (such as PRISMA extensions) to enhance reproducibility [66]. In summary, for URs to achieve robust application on a broader scale, further breakthroughs are needed in method standardization, dedicated tool development, and reporting transparency.

## 6 Methodology

The key steps in the development of URs include the following: First, clarifying research objectives and scope, defining research questions, and formulating the PICOS framework; second, systematically searching the literature, collecting published SRs/MAs through multiple databases and grey literature to reduce publication bias; third, setting strict inclusion and exclusion criteria, using tools such as AMSTAR‐2 to screen high‐quality studies and eliminate duplicate or low‐quality literature; fourth, extracting and organizing data, summarizing key information such as effect sizes and heterogeneity, while assessing the risk of evidence overlap; fifth, integrating conclusions using evidence synthesis methods, and grading the strength of evidence using systems such as GRADE; finally, presenting the results clearly in tables or graphs, discussing sources of heterogeneity and research limitations, and indicating future directions. The entire process requires transparency, typically involving pre‐registration of the protocol and adherence to standards such as PRISMA (Preferred Reporting Items for SRs and MAs).

### 6.1 Topic Selection

The selection of a research topic, which should possess clinical relevance, address current unresolved issues, and demonstrate both necessity and feasibility for undertaking URs, is fundamentally linked to the quality of UR development and subsequent result analysis. This initial step constitutes a critical prerequisite for the successful execution of URs. Therefore, when formulating research topics, several key factors must be systematically considered [38, 67]: (a) controversy: The selected topic should involve unresolved scientific debates or conclusions potentially influenced by unexamined systematic biases, as evidenced by conflicting or substantially divergent findings in existing SRMAs [10]; (b) clinical value: The topic's significance should be evaluated based on its potential to guide clinical practice or contribute to the development and revision of clinical guidelines, thereby providing a robust rationale for conducting URs; (c) data sufficiency: Prior to URs analysis, it is essential to verify the availability of adequate SRMAs data to ensure sufficient statistical power, minimize potential biases, and enhance the precision of estimates and interpretability of results. Following the topic selection, the research questions should be precisely defined using established frameworks such as PICOS [12, 68] or SPIDER (Sample, Phenomenon of Interest, Design, Evaluation, and Research type) [10, 69].

### 6.2 Inclusion and Exclusion Criteria

URs represent a comprehensive research methodology designed to systematically synthesize the existing evidence from SRMAs within a specific field. The establishment of inclusion and exclusion criteria is crucial to ensure the comprehensiveness and methodological rigor of the review process, which should be based on the research direction and characteristics, and this also serves as an additional response to the research topic and questions [70, 71]. The formulation of the inclusion and exclusion criteria should be guided by the PICOS framework [12]. The inclusion criteria are delineated as follows [72, 73, 74]: (a) study type: URs typically only incorporate SRMAs, as these studies have already synthesized and evaluated primary research. The inclusion of SRMAs mitigates redundant analysis of primary data while enhancing the efficiency of the evaluation; (b) study quality: Included SRMAs should possess high methodological rigor, usually assessed using validated tools such as AMSTAR; (c) research topic: Included SRMAs should align precisely with the specific research question or thematic focus of the URs, ensuring both relevance and methodological. The exclusion criteria are delineated as follows [12, 75, 76]: (a) non‐SRMAs: Primary studies, narrative reviews, and commentary articles are typically excluded due to their lack of systematic synthesis and comprehensive evaluation; (b) methodologically deficient SRMAs: Studies demonstrating inadequate methodological rigor, particularly those failing to explicitly delineate search strategies or conduct risk of bias assessments, were excluded; (c) thematically irrelevant SRMAs: Studies that were either entirely unrelated or only tangentially related to the research focus were excluded to maintain thematic coherence and analytical precision [75].

It is important to note the question of whether to include primary studies. Generally, URs do not directly incorporate primary studies, instead relying on existing SRMAs as their primary data sources. This approach not only reduces the analytical workload but also mitigates the potential for redundant analysis of primary data [77, 78]. However, in specific circumstances where a research field demonstrates a paucity of high‐quality SRMAs, the inclusion of rigorously selected primary studies may be justified. Such decisions must be supported by explicit rationale and implemented through stringent evaluation criteria to maintain methodological integrity [72, 73].

### 6.3 Registration

Similar to SRMAs, URs necessitate the pre‐registration of predefined inclusion and exclusion criteria in an SR repository such as the Prospective Register of SRs (PROSPERO). Researchers are required to comprehensively delineate the PICOS framework, along with the search strategy, database, data analysis methods, risk factors, bias assessment protocols, and definition of outcomes. The benefits of PROSPERO registration encompass four critical aspects: (a) documentation: PROSPERO registration ensures systematic recording of the research topic, thereby clarifying the study design and methodology, and facilitating subsequent operational processes; (b) bias mitigation: PROSPERO registration effectively prevents researchers from altering the original protocol or introducing human interference factors that could lead to selective reporting. By comparing the registered research plan with the final manuscript, potential selective bias can be identified; (c) duplication avoidance: PROSPERO registration allows researchers to upload their study concept to the database in advance, thereby preventing overlap with existing research direction and alerting others to avoid redundant resource expenditure; (d) enhanced credibility: Upon review and approved by the PROSPERO platform, a unique registration ID number is assigned to each research proposal. Certain journals mandate the inclusion of this ID number during manuscript submission, thereby significantly enhancing the credibility of the article and its likelihood of acceptance.

Although PROSPERO is the preferred database for multiple study registration, researchers may consider alternative registration platforms, such as the Open Science Framework (OSF). As a versatile open‐source platform, OSF offers distinct advantages for complex research projects requiring protocol modifications or interdisciplinary integration, particularly through its robust version control system and support for diverse file formats. However, it should be noted that OSF does not incorporate the peer review mechanism inherent to PROSPERO. Additionally, other viable registration platforms include the International Platform of Registered SR and Meta‐analysis Protocols (INPLASY), Zenodo, and Research Registry. The selection of an appropriate registration platform should be guided by specific research requirements and journal submission criteria. In certain cases, multi‐platform registration may be warranted to enhance research transparency and ensure methodological traceability.

### 6.4 Literature Searching and Screening

Prior to conducting literature retrieval, it is imperative to establish a rigorous and systematic search strategy to ensure the accuracy and comprehensiveness of the identified literature. The formulation of the search strategy should be guided by the PICOS framework and encompass the following key considerations [12, 79]: (a) search terms identification: A combination of Medical Subject Headings (MeSH) and free‐text terms should be employed, utilizing the MeSH database to ensure precise representation of the research topic. Additionally, synonyms, near‐synonyms, and related terms should be incorporated to enhance the sensitivity of the search; (b) databases selection: According to the research field and direction, research studies should select appropriate databases to search. Commonly used Chinese databases include CNKI, VIP, WanFang, and CBM, whereas prominent English databases comprise PubMed, Embase, the Cochrane Library, and Web of Science. Furthermore, unpublished gray literature should be identified through reference list screening or direct communication with authors [80]; (c) search strategy development: Boolean operators (AND, NOT, OR) should be strategically applied to combine search terms, thereby refining or expanding the search scope to improve precision and recall; (d) specification of literature types: Search terms derived from SRMAs or those recommended by the Scottish Intercollegiate Guidelines Network (SIGN) should be utilized to enhance the specificity of the search; (e) assessment of search outcomes: The retrieved results should be evaluated against predefined inclusion and exclusion criteria, with subsequent adjustments to the search strategy as necessary to ensure the relevance of the included literature to the research topic.

The final retrieved literature is systematically imported into reference management software (Endnote, Mendeley, Zotero, or NotExpress) or online platforms (Covidence or Rayyan) to optimize the efficiency of screening process [81, 82, 83]. The literature screening procedure is conducted through a structured approach comprising the following key steps: (a) duplicates removal: Identical records across multiple databases are identified and eliminated using the deduplication functionality of the reference management software. In cases where multiple SRMAs address the same research topic, the most recent publication is prioritized [12]; (b) preliminary screening: Researchers perform an initial assessment based on predefined inclusion criteria, evaluating titles and abstracts to identify potentially relevant studies; (c) full‐text evaluation: Articles are thoroughly reviewed in accordance with the PICOS framework to ensure methodological relevance and alignment with the research objectives; (d) literature classification: Studies are systematically categorized based on predefined criteria, including duplicates, non‐SRMAs, methodologically flawed studies, and inaccessible full‐text articles, to facilitate the generation of a PRISMA‐compliant flowchart. To ensure methodological rigor, the screening process is independently conducted by at least two rigorously trained evaluators, with subsequent cross‐verification to minimize classification errors and enhance the reliability of the included studies [12, 84]. Any discrepancies between evaluators are resolved through consensus‐based discussion or consultation with a third independent reviewer.

### 6.5 Data Extraction

Following the identification of eligible literature for inclusion, a systematic data extraction process must be conducted to obtain essential information for subsequent quantitative and qualitative analyses. This critical step serves as the foundation for successful URs, as it directly influences the subsequent analytical procedures and result interpretation. To ensure methodological rigor, it is strongly recommended that two independent reviewers perform duplicate data extraction, with cross‐verification to ensure accuracy and reliability [39]. A standardized data extraction form should be developed in advance, enabling both reviewers to independently extract relevant qualitative and quantitative data.

The qualitative data elements include primary author, publication year, study design (such as RCTs), demographic characteristics (sex, region/ethnicity), intervention details, effect model specification (random/fixed‐effects), and potential bias assessment. The quantitative data elements include number of included studies, participant age characteristics (mean and range), total sample size, experimental/control group sample size, and effect size measures (relative risk (RR), odds ratio (OR), or mean difference) with corresponding 95% confidence intervals.

It is worth noting that two critical considerations must be addressed during data extraction: (a) For SRMAs incorporating multiple effect endpoints, each endpoint requires individual extraction; (b) when included studies demonstrate methodological limitations or insufficient quantity, supplementary extraction of high‐quality primary study data is essential to minimize bias and enhance the overall quality of URs [12]. The final data should preferentially derive from the most recent or the largest population‐based studies. Any discrepancies in extracted data should be resolved through consensus or adjudication by a third reviewer to ensure data integrity.

### 6.6 Methodological Quality Assessment

In URs, whether to assess the quality of primary studies typically depends on the specific objectives and methodological design of the research. Generally, URs primarily integrate evidence based on published SRMAs, with the focus of evaluation being on the methodological quality of the SRMAs themselves, rather than directly re‐evaluating the primary studies. This is because the primary studies have usually already undergone quality assessment within the included SRMAs [62, 64]. It is reasonable and effective to directly adopt the results of SRMAs when they are of high methodological quality, comprehensively cover the research question, have transparent data extraction and analysis processes, and exhibit low levels of evidence overlap [24, 37]. However, if SRMAs have significant limitations (such as omitting key studies or having biased analysis methods), or if the research objectives require a more in‐depth risk of bias analysis of the evidence base, it may be necessary to trace back and re‐evaluate the quality of the primary studies [21, 85]. In summary, URs should prioritize comprehensive inference on the basis of high‐quality SRMAs. Only when there are significant gaps in the evidence, serious contradictions in the conclusions, or a need to further verify the robustness of the results should supplementary quality assessments of primary studies be considered. Whether to conduct such in‐depth assessments requires a comprehensive judgment based on the specific objectives of the URs, the quality and completeness of the available evidence, and the research resources [65, 86, 87].

Given that the quality of URs largely depends on the quality of the included studies, it is strongly recommended that at least two researchers independently assess the methodological quality and confidence of evidence of the included studies before conducting URs to ensure methodological rigor and credibility of the evidence. Current quality assessment methodologies predominantly employ established tools such as AMSTAR‐2 [88], the Risk of Bias in SR (ROBIS) [89], and specialized tools like the Critical Appraisal Tool for Anatomical Meta‐analysis (CATAM) [90]. These tools are strongly recommended for their extensive validation and application in SRMAs, and their methodological congruence renders them equally suitable for URs [91, 92].

AMSTAR‐2, as a widely utilized assessment tool, consists of 16 entries across 7 key domains, providing a comprehensive evaluation framework for SRs [88]. Its advantages lie in its emphasis on core methodological elements, including adherence to PICOS principles, the thoroughness of the search strategies, and the appropriateness of data analysis. The tool employs a quantitative scoring system (1 point per criteria met), facilitating straightforward quality comparisons among studies. However, AMSTAR‐2 exhibits certain limitations, such as a superficial assessment of risk of bias, rigid scoring criteria, and limited adaptability to novel methodologies like network meta‐analysis [93].

Conversely, ROBIS is specifically designed to evaluate the risk of bias in SRMAs [89]. This tool employs a three‐phase assessment process (association assessment, identification of risk of bias, and overall risk of bias assessment), focusing on critical aspects of the research process that may introduce bias. The advantage of ROBIS lies in its applicability to all SRMA types, including diagnostic test accuracy reviews. Nonetheless, its evaluation process is complex, demands a high level of evaluator expertise, and lacks a quantitative scoring system, making direct comparison among different studies difficult [94, 95].

In practice, a stepwise evaluation strategy is recommended [96]. Initial rapid screening of included literature using AMSTAR‐2 should focus on seven key areas: adherence to PICOS principles, clarity of inclusion and exclusion criteria, comprehensiveness of search strategies, appropriateness of data analysis, thoroughness of risk of bias assessment, management of conflicts of interest, and completeness of results reporting. Subsequently, the ROBIS tool should be employed to assess the risk of bias, emphasizing the clarity of research questions, comprehensiveness of literature retrieval, standardization of data extraction, scientific rigor of results synthesis, and rationality of conclusions. The combined results from both tools can classify studies into three quality grades: high (meeting both AMSTAR‐2 high score and ROBIS low risk criteria), medium (meeting one of the criteria), and low (failing to meeting either criteria). This integrated evaluation approach not only guarantees the comprehensiveness of the assessment but also facilitates the effective identification of potential bias risk, thereby establishing foundation for subsequent evidence synthesis. It is crucial to emphasize that researchers must undergo comprehensive training and implement consistency testing before utilizing these assessment tools to ensure the reliability and reproducibility of the assessment outcomes.

### 6.7 Methodological Evidence Assessment

The methodological quality of the included studies was assessed using the aforementioned approaches. Concurrently, it is also essential to evaluate the confidence in the evidence derived from these studies, typically through a modified GRADE [97, 98]. Although the GRADE system was originally developed for individual SRs, it can also be applied to the URs with appropriate modifications [99, 100]. Key considerations in this evaluation include the following: (a) The study design, which significantly impacts the quality of evidence, with RCTs generally provides high‐quality evidence, whereas observational studies are more susceptible to confounding factors and thus yield low‐quality evidence; (b) the rigor of study implementation, encompassing the adequacy of study design, application of blinding, management of loss to follow‐up, and the assessment of publication bias; (c) the evaluation of evidence quality is contingent upon three critical dimensions: consistency (the degree of concordance in findings across diverse studies), directness (the extent to which study outcomes are clinically applicable), and precision (the sufficiency of sample size and magnitude of effect estimates). These factors collectively inform the robustness and reliability of the evidence synthesis. Additionally, factors that may enhance the credibility of evidence, such as the presence of a dose–response relationship, a large effect size, and the control of negative bias, should be considered.

However, the application of GRADE to URs presents certain limitations [101]. First of all, URs synthesize SRMAs rather than primary studies, necessitating adjustments the traditional GRADE criteria. Furthermore, the reliability of GRADE ratings may be compromised by methodological heterogeneity among the included SRMAs. Therefore, it is recommended to rigorously assess the methodological quality of the included SRMAs, account for the degree of heterogeneity among them, and integrate other evidence classification criteria to form a comprehensive judgment. Here we present two common evidence grading methods: the modified GRADE four‐level classification system and the five‐level evidence classification criteria [102, 103]. The modified GRADE four‐level classification categorizes evidence as follows: high (all key criteria met with no more than one non‐key criterion deficient); medium (minor deficiencies, all key criteria met but more than one non‐key criterion deficient); low grade (significant deficiencies, one key criterion unmet, including/excluding non‐key criteria); and very low (serious deficiencies, more than one key criteria unmet, including/excluding non‐key criteria) [104]. According to international standards, the quality of evidence can be divided into five categories: I (convincing evidence); II (highly suggestive evidence); III (suggestive evidence); IV (weak evidence); and V (insufficient evidence). For Categories I and II evidence, sensitivity analyses can be further performed to verify the stability of the results [25, 105, 106, 107].

### 6.8 Data Analysis

Data analysis constitutes a fundamental component of URs. Through comprehensive mining and rigorous analytical methodologies applied to the study dataset, researchers can obtain a more holistic and objective understanding of the relationship between the intervention measures and the outcomes effect, thereby providing robust evidence for subsequent outcome evaluation and evidence‐based decision‐making.

To enhance analytical efficiency, we recommend utilizing the R programming language in conjunction with the metaumbrella package for statistical analysis [43]. The metaumbrella represents the first comprehensive statistical suite specifically developed for URs, providing a complete analysis workflow from data preprocessing to evidence stratification. This package effectively addresses common methodological challenges in URs, including the data transformation and effect size standardization, significantly improving analytical efficiency and methodological rigor.

The data analysis encompasses three primary methodological stages: (a) data preprocessing, wherein metaumbrella package's integrated data cleaning functionalities are employed, including outlier detection through established algorithms (such as boxplot and standard deviation methods), missing data handling via multiple imputation techniques, and automated correction of common data entry errors; (b) effect size standardization, facilitated by the package's comprehensive conversion algorithms that ensure cross‐study comparability, including transformations between categorical data metrics (OR, RR, risk difference (RD)), standardization of correlation coefficients (Pearson's *r*, *R* ^2^, etc.), and equivalence conversions among difference metrics (Cohen's *d*, Hedges's *g*), with specific conversion formulas detailed in Table 2 [11, 108]; (c) descriptive statistical analysis: generating comprehensive distributional characteristics, through central tendency measures (mean, median), dispersion metrics (standard deviation, interquartile range), and range indicators (minimum, maximum). The metaumbrella package's unique capabilities for URs include an automated evidence grading system (Categories I–V), integrated sensitivity analysis, advanced visualization tools, and support for MAs of multiple effect sizes, thereby enhancing efficiency, accuracy, reproducibility, and transparency. Additionally, the metaConvert package offers complementary functionalities, including automated calculation of 11 distinct effect size metrics, innovative handling of overlapping datasets, comparative analysis of dataset variations, and management of effect size dependencies, significantly improving the reliability and efficiency of effect size estimation [109]. These tools are particularly valuable for researchers conducting MAs as well as for any investigators requiring robust methods for effect size estimation.

**TABLE 2:** Effect sizes and their conversion formula.

Effect size	Conversion formula and explanation
**Categorical family (OR‐family): It is suitable for binary data**	
OR	It is an effect size, which is used to compare the probability of an event occurring in two groups, including case–control and cohort studies Equations derived from the definitions of OR and RR: $\mathrm{OR}\;=(\frac{1-P_{0}}{1-P_{1}})\times \mathrm{RR}$ Researchers need to assess the event rates (*P*_0_ and *P*_1_) in the control and exposed groups. When *P*_0_ and *P*_1_ are sufficiently small, RR ≈ OR
RR	It is an effect to measure of risk, which is used to compare the difference in the probability of an event occurring in the control and exposed groups and is also the most useful indicator for the strength of the association between exposure and morbidity. RR = 1: no statistical association between exposure factors and outcome RR > 1: positive association between exposure factors and outcome RR < 1: negative association between exposure factors and outcome
RD	It is an effect size for assessing risk, which represents the absolute difference in the probability of an event occurring between the control and exposed groups Equations derived from the definitions of RD and RR: $\mathrm{RR}\;=1+\frac{1}{P_{0}}\times \mathrm{RD}$
**Correlation family (*r*‐family): It is suitable for correlational data**	
Pearson's *r*	A correlation coefficient, which measures the strength of the linear relationship between two variables. Close to 1 indicates a strong positive correlation Close to −1 indicates a strong negative correlation Close to 0 indicates no linear relationship Converting Pearson's *r* to approximate Cohen's *d*: $d=\frac{2\times r}{\sqrt{1-r^{2}}}\;$
*R* ^2^ (*r*‐squared)	*R* ^2^ is the ratio of the regression sum of squares to the total sum of squares, which represents that the model can explain the proportion of total variation
*η* ^2^ (Eta‐squared)	*η* ^2^ is used to measure the proportion of variation about dependent variable and independent variable in ANOVA The value of *η* ^2^ ranges from 0 to 1. Larger values indicate that the independent variable explains more of the variation in the dependent variable
*ω* ^2^ (omega‐squared)	*ω* ^2^ is a modified effect size, which is used to measure the proportion of variation about the dependent variable and the independent variable in analysis of variance (ANOVA) The value of *ω* ^2^ also ranges from 0 to 1, but it usually gives a more conservative estimate of the effect size than *η* ^2^
**Difference family (*d*‐family): It is suitable for continuous data**	
Cohen's *d*	It is an effect size to measures the difference in means, which is used to compare the difference in means between two groups There are three effect sizes, small, medium, and large, corresponding to standard deviation units of 0.2, 0.5, and 0.8, respectively
Hedges's *g*	It is a corrected form of Cohen's *d*, Equations derived from the definitions of Hedge's g and Cohen's *d*: $d=g\times \frac{1}{J(\mathrm{df})}$ when the sample size is large enough, the correction factor (*J*) is about 1, *g *≈ *d*

Abbreviations: OR, odds ratio; RD, risk difference; RR, risk ratio.

#### 6.8.1 Heterogeneity Analysis

Heterogeneity analysis, as a critical component in evaluating discrepancies among the outcomes of various studies, aids researchers and readers in comprehending the underlying reasons for the observed discrepancies. To mitigate the limitations associated with a single metric, it is advisable to employ a combination of complementary methods for a comprehensive assessment. Currently, there are three widely utilized approaches for assessing heterogeneity, including the *I* ^2^ statistic, the *Q* statistic (Cochrane's *Q* test), and the *H* statistic (Kruskal–Wallis *H* test) (Supporting Information A) [110, 111, 112].

The *I* ^2^ statistic quantifies the extent of heterogeneity across studies, with values ranging from 0% to 100%. It is essential to recognize that *I* ^2^ is not an absolute measure of heterogeneity, and its interpretation should be contextualized with confidence intervals rather than relying on fixed thresholds [113]. It is recommended to report *I* ^2^ with 95% confidence intervals. Higher *I* ^2^ values indicate greater heterogeneity among studies, and the interpretation of the *I* ^2^ statistic typically categorizes heterogeneity into four distinct levels [39]. In practice applications, the *I* ^2^ value can guide the selection of an appropriate effect model. For instance, a fixed‐effects model may be appropriate when the *I* ^2^ value is low (e.g., <25%), whereas a random‐effects model may be more suitable when the *I* ^2^ value is high (e.g., >50%). However, the *I* ^2^ value is not the sole determinant in selecting the effect model. Researchers must also consider other factors, such as study design, sample size, and study quality [114, 115].

The *Q* statistic serves as a methodological tool for evaluating heterogeneity among multiple independent studies, following a chi‐square distribution, where higher value indicates greater inter‐study variability, thus reflecting stronger heterogeneity. Statistical significance of heterogeneity was determined using the chi‐square test, with a predefined significance threshold of *p *< 0.10 [116, 117]. It is important to note that the statistical power of the *Q* test is substantially influenced by the number of studies included in the analysis.

In contrast, the *H*‐statistic, derived from the Kruskal–Wallis test, assesses difference between medians of multiple independent samples and is adjusted for degrees of freedom (df), making it less susceptible to changes in the number of studies [118]. Supporting Information A details the methodologies for heterogeneity assessment. Both the *H*‐statistic and *I* ^2^ statistic employ degrees of freedom to mitigate the impact of study number on the *Q* value, resulting in more stable and reliable heterogeneity outcomes [119]. However, the *Q* test is particularly sensitive to the number of included studies and focuses solely on sample number without considering sample quality. In cases of significant heterogeneity, it is imperative to elucidate and investigate the sources of such variability. Consequently, model selection should not rely exclusively on the *I* ^2^ value but should incorporate a comprehensive evaluation of clinical and methodological similarities among studies, heterogeneity test results, and the anticipated distribution characteristics of effect sizes. This multi‐dimensional approach to heterogeneity assessment provides a more robust foundation for the interpretation of results. All analytical procedures can be executed within the R environment utilizing the metafor and metaumbrella packages to ensure methodological rigor [43, 120].

#### 6.8.2 Bias Assessment

Following the heterogeneity analysis, a rigorous assessment of potential biases in the research data is imperative. The methodological evaluation utilizes four established risk‐of‐bias assessment tools (detailed in Table S4): (a) the Newcastle Ottawa Scale (NOS); (b) the Cochrane risk of bias tool; (c) the Quality Assessment of Diagnostic Accuracy Studies 2 (QUADAS‐2); (d) the JBI tool [121]. The aforementioned methods can also be utilized to indirectly assess the included data, including AMSTAR‐2, ROBIS, and GRADE.

Publication bias was often examined using Egger's method, which evaluates the relationship between effect sizes and standard errors in MAs, with a significance threshold of *p *< 0.10 indicating a higher probability of publication bias [122]. To enhance the robustness of this assessment, multiple complementary methods were employed, such as funnel plot analysis and Begg's test, ensuring comprehensive evaluation of the included studies’ reliability [123]. In instances where the included SRMAs demonstrate evidence of reporting bias, it is imperative to meticulously extract pertinent data elements on an individual basis and comprehensively delineate the underlying mechanisms contributing to such bias. Furthermore, it may be warranted to conduct a reincorporation and re‐analysis of the primary studies to ensure methodological rigor.

#### 6.8.3 Sensitivity Analysis

Sensitivity analysis represents a critical statistical method employed to evaluate the robustness of model outputs or analytical results in response to variations in certain key parameters. Within the context of URs, this approach is frequently utilized to determine the stability of SRMAs, particularly regarding their consistency across diverse analytical methods and nadir criteria. The following four principal assessment modalities are typically implemented [80]. (a) Sensitivity coefficients: Quantify the responsiveness of analytical indicators to parameter uncertainties; (b) critical point analysis: Identify threshold conditions at which analytical outcomes undergo significant alterations; (c) graphical representation: Visual depictions of sensitivity analysis results are generated through various graphical formats, including histograms, box‐and‐whisker plots, and line graphs, to illustrate differential outcomes under varying analytical conditions; (d) multivariate sensitivity analysis: When warranted, this comprehensive approach can be conducted to assess the cumulative impact of simultaneous variations in multiple parameters on the analytical results.

In conducting the sensitivity analysis of URs, the researchers must meticulously document each procedural step, including methodological approaches, parameter configurations, and resultant variations, to ensure reproducibility and facilitate independent verification. This documentation enables comparative assessment of evidence rankings against prior evidence grading systems, with necessary recalibration if discrepancies are identified [12]. In addition, the results of sensitivity analyses should be explicitly reported in the study to provide readers with a comprehensive understanding of the analytical limitations of and the reliability of the derived conclusions.

### 6.9 Overlap of Included Studies

URs encompass secondary studies rather than primary studies, which introduces the potential for data overlap and subsequent bias in the outcomes of URs when multiple SRMAs incorporate the same primary study [80, 124].

To mitigate the issue of data overlap, several strategies can be employed: (a) During the collection and screening of SRMAs, reference lists of different SRMAs should be cross‐referenced to identify overlapping studies and their frequencies utilizing literature management software or manual verification; (b) for two or more overlapping studies, the appropriate study data should be selected based on criteria such as the recency of the literature, the largest number of primary studies, and the strongest relevance of the research question [11]. Additionally, researchers can utilize the corrected covered area (CCA) formula to quantify study overlap and compare the results to select the most appropriate study data [125, 126, 127].

The CCA serves as a statistical tool to quantify and evaluate the extent of study overlap among the included SRMAs [128, 129]. The calculation of CCA involves the following steps (Supporting Information 3): (a) defining the scope of SRMAs: This entails determining the number of studies included and their specific characteristics within each SRMAs; (b) constructing matrices: The rows and columns of the matrices represent, respectively, the number of retrieved primary studies and SRMAs to be compared. Each cell in the matrices indicates whether each particular primary study is included in SRMAs (typically represented by 1 for inclusion and 0 for exclusion); (c) calculating the CCA: The formula for calculating the CCA is as follows:

$$\mathrm{CCA}\;=\frac{N-r}{\left(r\times c\right)-r}\times 100\%$$

where the CCA is quantified as a percentage [129], where *N* represents the total number of primary studies included in the evidence synthesis (including duplicates, as indicated by the selected boxes in the citation matrices); *r* denotes the number of rows, representing the number of primary studies retrieved for publication; and *c* denotes the number of columns, representing the number of SRMAs to be compared.

We present an example of how to calculate the CCA in Supporting Information B. According to the CCA value, the overlap of studies can be classified into four levels: very high‐overlap (>15%), high‐overlap (11%–15%), medium‐overlap (5%–10%), and low‐overlap (0%–5%) [128]. It is generally considered that CCA > 10% suggests substantial overlap among the included studies, necessitating further evaluation of the quality of the SRMAs and the primary study data to assess analytical methodologies and mitigate potential biases and limitations. Conversely, the CCA ≤ 10% suggests minimal overlap, requiring retention and comparison of analytical data from among studies to assess their impact on the results [130]. However, even when the CCA ≤ 10%, bias and error may arise due to human factors, such as sampling discrepancies resulting from variations in study topics, inclusion and exclusion criteria, or search strategies. Consequently, the study inclusion must strictly adhere to the study topic and predefined criteria. In cases of narrow study scope, additional qualitative assessment is warranted to explain potential reasons for low‐overlap [129, 131].

The CCA serves as a preliminary assessment tool for evaluating study overlap in URs [132, 133]. The selection of overlapping studies is typically conducted based on the following hierarchical criteria: (a) methodological quality, assessed using the AMSTAR‐2 tool, with inclusion restricted to studies meeting at least medium quality standards; (b) temporal relevance, prioritizing studies with more recent publication dates; (c) analytical approach, requiring included studies to provide pooled effect estimates of SRMAs data; and (d) sample size, preferentially selecting studies with larger participant populations to minimize potential confounding bias. However, the CCA formula has not been universally adopted, and researchers often adapt their approach to overlapping studies on the basis of specific research objectives [134], such as employing weighted CCA to quantify the degree of information overlap between SRMAs [135].

To address the challenge of data overlap in SRMAs within URs, we strongly recommend for the implementation of the CCA in R (CCAR) toolkit, which facilitates quantitative assessment and visual analysis of study overlap [136]. The R package offers a comprehensive solution to assess the extent of primary study overlap among SRMAs, featuring automated calculations, visual analysis, and overlap threshold determination. To mitigate bias, it is strongly recommended that SRMAs exhibiting substantial overlap (CCA > 10%) should be excluded during the literature screening phase, with the extent of overlap explicitly reported in the results section. In cases where substantial overlap is unavoidable (e.g., when the included studies are of landmark significance but exhibit overlap), researchers should thoroughly discuss its potential impact on pooled effect estimates and conduct sensitivity analyses (e.g., recalculating effect sizes after excluding highly overlapping SRMAs) to evaluate result robustness. Furthermore, when exclusion of overlapping studies is not feasible, advanced statistical techniques, such as generalized linear mixed models (GLMM) [137, 138] or multilevel meta‐analysis [42, 139], should be employed to adjust for overlap effects, thereby enhancing the reliability of evidence synthesis.

### 6.10 Updating Existing Evaluation Studies

The URs mainly incorporate SRMAs rather than primary studies. Therefore, the currency of the included study types constitutes a critical step in URs, directly influencing the reliability and generalizability of URs outcomes [36, 67]. To assess the necessity of updating the included SRMAs, a structured literature review framework can be employed, which comprises two sequential steps [24, 140]. (a) Sorting: Newly published studies are categorized in descending order based on sample size; (b) merging: The effect estimates of the newly published studies are sequentially integrated with the overall effect estimates of the SRMAs to evaluate the currency of the original SRMAs. An SRMA is deemed outdated if the incorporation of newly published studies resulted in either a change in statistical significance or a shift exceeding 50% in relative effect sizes.

It is worth noting that when screening data from studies, the prioritization of updates can be determined through a comprehensive assessment of three factors: the proportion of key questions requiring updates, the urgency of updating specific conclusions, and the degree of obsolescence [141]. The SRMAs studies excluded from URs are usually classified into three categories: (a) definitely outdated, (b) probably outdated, and (c) still valid. For the initial two categories, prioritization of updates should be guided by the following hierarchical criteria: (a) incorporation of recently published studies demonstrating substantial scholarly impact, such as multiple citations, or meeting the quality threshold of moderate or higher based on AMSTAR‐2 assessment tool; (b) systematic identification of newly published studies that satisfy the predefined inclusion criteria for URs; (c) evaluation of the potential impact of newly identified studies on the robustness, validity, or directionality of existing SRMA conclusions.

### 6.11 Visualization and Reporting of Results

**FIGURE 2:**
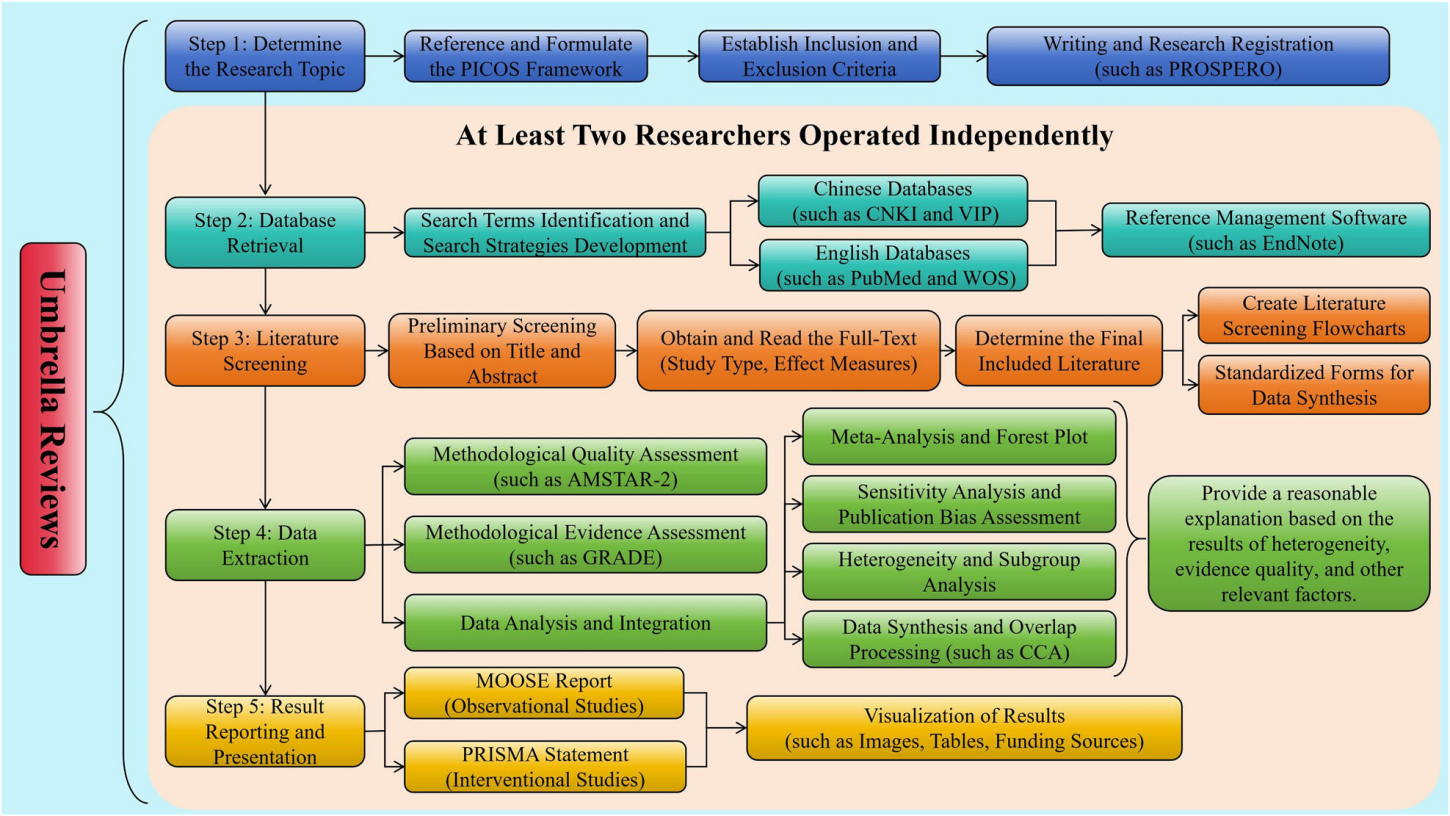
Umbrella analysis flowchart. AMSTAR‐2, A Measurement Tool to Assess Systematic Reviews 2; CCA, corrected covered area; GRADE, Grading of Recommendations Assessment, Development, and Evaluations; MOOSE, Meta‐analysis Of Observational Studies in Epidemiology; PICOS, Patient/Population, Intervention, Comparison, Outcome, Study design; PRISMA, Preferred Reporting Items for Systematic Reviews and Meta‐Analyses; PROSPERO, The International Prospective Register of Systematic Reviews; WOS, Web of Science.

URs mainly rely on pooled effect sizes derived from SRMAs, with their methodological framework and reporting standards guided by established guidelines such as PRISMA [142, 143, 144], Meta‐analysis Of Observational Studies in Epidemiology (MOOSE) [12], and Preferred Reporting Items for Overviews of Reviews (PRIOR) [145, 146]. The reporting structure encompasses seven key components (Figure 2): (a) determine the research topic, which can reference and formulate the PICOS framework, including the inclusion and exclusion criteria, to facilitate advance registration and reduce the likelihood of duplicating studies; (b) database retrieval, detailing description of the search strategies, including databases and search terms; (c) literature screening, capturing study characteristics such as authors, publication year, study design, population, interventions or exposures, outcomes, sample sizes, and effect sizes; (d) methodological quality and evidence assessment; (e) data analysis, including heterogeneity and sensitivity analysis; (f) data synthesis and overlap processing; and (g) result reporting and presentation, including literature screening flowcharts, bias risk plots, and forest plots, to enhance the clarity and interpretability of results.

It should be noted that several critical considerations must be addressed when reporting the URs: (a) data description: The reporting of URs should explicitly indicate whether the included studies adhered to established guideline processes; when primary study data are incorporated, these should be individually extracted and clearly described; (b) endpoints description: The endpoints of URs should be reported with maximal detail and comprehensive, ensuring clarity and intuitive presentation. Heterogeneity among included studies should be addressed through subgroup analysis or other methods, with a thorough explanation of differences and relationships between subgroups and outcomes from multiple perspectives [12]; (c) description of bias: Bias reporting should encompass traditional considerations, including absolute risk reduction, reverse causality, selection bias, and information bias. Additionally, a causal analysis should be conducted on the basis of the included literature to assess the relationship between the research question and potential causal factors; (d) other descriptions: The report should explore other potential factors to the results and provide appropriate extrapolation of the study conclusions within the scope of the research question. Ideally, these conclusions should be appropriately extrapolated, preferably providing guidance for future research on the research question. Finally, ethical issues and other information, such as the funding sources of the studies, should be disclosed [147].

## 7 Discussion

The URs represent an advanced tertiary study methodology that synthesizes evidence from SRMAs [68, 92]. Although URs share methodological similarities with SRMAs, they offer superior levels of evidence and demonstrate enhanced comprehensiveness and instructional value. This approach enables researchers to efficiently organize and analyze relevant data and findings pertaining to specific study topic, thereby significantly optimizing the literature search and screening process. The implementation of URs facilitates the critical evaluation of methodological strengths and limitations across studies while providing readers with a comprehensive conceptual framework of research domain. Furthermore, the utilization of URs offers valuable methodological frameworks and guidance for the SRs and critical evaluation of analogous studies in subsequent research endeavors [148, 149].

This study aligns with the methodological best practices by Belbasis et al., which recognize URs as a critical methodological approach for synthesizing SRMAs to address evidence fragmentation [12]. Both approaches emphasize systematic literature search protocols, dual independent screening, and rigorous quality assessment utilizing GRADE. Simultaneously, the current study advances these established procedures through three methodological enhancements: (a) Implementation of an enhanced approach to methodological heterogeneity assessment through a multi‐dimensional evaluation strategy incorporating *I* ^2^, *Q*, and *H*‐statistics, contrasting with the basic heterogeneity assessment proposed by Belbasis et al. [12]; (b) introduction of specialized statistical tools, including the metaumbrella package for automated effect size standardization and the CCAR toolkit for quantitative overlap analysis, thereby addressing technical implementation gap in the framework of Belbasis et al.; (c) establishment of quantitative criteria for updating SRMAs, defining “outdated” reviews as those demonstrating a >50% change in relative effect sizes following the incorporating of new evidence, whereas Belbasis et al. provided only general guidelines. Additionally, although the Belbasis et al. primarily focused on medical and epidemiological applications, this study demonstrates the broader utility of URs across educational and public health domains. We sincerely appreciate the contributions of Belbase et al., who have laid a clear and solid foundation for EBM practice. It is based on their pioneering work that we have been able to further refine the details by introducing more rigorous quality control processes and interdisciplinary adaptation tools, thereby expanding the boundaries the original methods. These supplements do not diminish, but rather respect and extend the contributions of Belbasis et al., with the expectation of infusing new vitality into this method in more clinical scenarios and other fields [12].

Meanwhile, this study significantly advances methodological framework for the data analysis in URs through three pivotal innovations. First, it systematically incorporates the metaumbrella package in R, facilitating automated preprocessing (e.g., outlier detection via boxplot methods and multiple imputation for missing data) and standardized conversion of effect sizes (e.g., automatic conversion between OR, RR, and RD, as well as equivalent transformation of Cohen's *d* and Hedges's *g*). This integration significantly enhances the efficiency and consistency of cross‐study comparisons [43, 120]. Second, the study introduces a combined approach utilizing CCA and GLMM to address data overlap among included SRMAs. The CCA quantifies overlap levels (with a threshold of >10% indicating high overlap), whereas GLMM provides statistical correction when high overlap is unavoidable, thereby mitigating bias arising from repeated primary study data [135, 137]. Third, the study introduces the metaConvert package to refine effect size estimation, enabling flexible calculation of eleven effect size metrics, managing overlapping input data, and resolving dependencies among effect sizes, thereby improving the reliability of pooled results [109]. These innovations not only refine the methodological rigor of URs but also offer practical tools for researchers to handle complex data scenarios.

This article provides a systematic and comprehensive introduction to the concept and critical characteristics of URs, with a particular focus on their data analysis and methodology requirements. Additionally, it also compares URs with the secondary research and discusses both the value and current challenges of conducting such reviews, aiming to facilitate their broader application in future evidence‐based practice. In summary, URs serve as an important tool for integrating evidence, guiding policy, and identifying research gaps across various disciplines. Although still under ongoing development and refinement, continued theoretical advances and deeper methodological rigor will enable URs to offer a scientific, comprehensive, and optimal evidence base, as well as innovative theoretical frameworks for clinical research and health management decision‐making in the future.

## Funding

This work was supported by the Scientific Research Fund Project of Yunnan Provincial Department of Science and Technology (No. 202101AT070234) and the Scientific Research Fund Project of Yunnan Provincial Department of Education (No. 2024Y227).

## Conflicts of Interest

The authors declare no conflicts of interest.

## Supporting information




**Table S1**: Interpretation of I‐statistic Results
**Table S2**: Interpretation of Q‐statistic Results
**Table S3**: Interpretation of H‐statistic Results
**Table S4**: Common Tools for Assessment Bias
**Table S5**: Matrices of SRMAs
**Table S6**: CCA Calculation Example
