# Supplementary material for: Umbrella Reviews: Concepts, Methodological Frameworks, and Step‐by‐Step Implementation
Source: J Evid Based Med. 2025 Dec 16;18(4):e70092. doi: 10.1111/jebm.70092 (PMC12750496; doi:10.1111/jebm.70092)
Supplement: Supplementary file 1 — Table S1: Interpretation of I‐statistic Results Table S2: Interpretation of Q‐statistic Results Table S3: Interpretation of H‐statistic Results Table S4: Common Tools for Assessment Bias Table S5: Matrices of SRMAs Table S6: CCA Calculation Example [file JEBM-18-0-s001.docx]

**Supplementary Materials**

**Supplementary Material A. Heterogeneity Analysis**

1. **The I² statistic**
2. Purpose:assesses the difference in effect sizes among studies as a precentage of total difference;
3. Calculation formula:$I^{2}=100\%*\frac{Q-df}{Q}$ , where Q is the value of the Q statistic and df is the number of literature included-1;

**Table S1. Interpretation of I-statistic Results**

| **Categorisation** | **Heterogeneity** | **Interpretation of results** | **Effect model** |
| --- | --- | --- | --- |
| 0% - 40% | Low-degree | Less variation among studies | I² >50%:Adoption of a random effects model |
| 30% - 60% | Medium-degree | Some variation among studies |  |
| 50% - 90% | High-degree | Wide variation among studies | I² <50%:Adoption of the fixed effects model |
| 75% - 100% | Very High-Degree | very large variation among the studies |  |

1. **The Q statistic**
2. Purpose:assesses the standardized weighted sums of squares among variation of studies;
3. Calculation formula:$Q=\sum_{i=1}^{k} \left[ wi*\left( {ES}_{i}-ES \right)^{2} \right]$, k is the number of studies; wi is the weight of the ith study; ESi is the effect size of the ith study; and ES is the weighted average of the effect sizes of all studies;

**Table S2. Interpretation of Q-statistic Results**

| **Level of significance** | **Interpretation of results** |
| --- | --- |
| P> 0.1 | Heterogeneity among studies |
| P< 0.1 | No heterogeneity among studies |

1. **The H statistic**
2. Purpose:Assess whether there are differences in measurement or hierarchical information across multiple independent samples;
3. Calculation formula:$\text{H=}\sqrt{\frac{\text{Q}}{\text{df}}}$ , where Q is the value of the Q statistic and df is the number of literature included-1;

**Table S3. Interpretation of H-statistic Results**

| **Heterogeneity** | | **Level of Significance** | **Interpretation of Results** |
| --- | --- | --- | --- |
| Value of H < 1.2 | | P < 0.05 | No heterogeneity among studies |
| 1.2< Value of H <1.5 | The 95% confidence interval contains 1 |  |  |
|  | The 95% confidence intervals do not contain 1 |  | Heterogeneity among studies |
| Value of H >1.5 | |  |  |

**Table S4. Common Tools for Assessment Bias**

| **Tools** | **Explanation** |
| --- | --- |
| NOS | For assessing the quality of observational studies, including literature selection, comparability and outcome assessment. Each section has a number of entries, which are rated by the researcher according to how well they are met, with higher ratings indicating better research quality |
| Cochrane Risk of Bias Assessment tool | Dedicated to assessing the risk of bias in randomized controlled trials, including implementation of the experiment, completeness of outcome data, etc. A series of questions are used to assess the potential risk of bias of RCTs during the design, implementation and reporting phases and to provide an overall assessment of the risk of bias. |
| QUADAS-2 | For assessing the risk of bias in diagnostic tests, including aspects of patient selection, trial conduct, outcome assessment, and trial flow.  Assessing diagnostic trial studies, including the design, quality of conduct and interpretation and application of results through a series of entries and providing a comprehensive assessment of bias risk. In response, researchers can identify and interpret the reliability of study results. |
| JBI | It provides a range of tools and resources to support evidence-based practice and research. |

NOS, Newcastle-Ottawa Scale; QUADAS-2, Quality Assessment of Diagnostic Accuracy Studies - 2; RCTs, Randomized Controlled Trials; JBI, Joanna Briggs Institute.

**Supplementary Material B. Calculate the Overlap Between Included Studies**

**Table S5. Matrices of SRMAs**

| **Primary studies included（r）** | **Comparative SRMAs needed（c）** | | |
| --- | --- | --- | --- |
|  | **A1** | **A2** | **A3** |
| A | 1 | 1 | 0 |
| B | 0 | 1 | 1 |
| C | 1 | 0 | 1 |
| D | 0 | 1 | 0 |
| E | 1 | 1 | 0 |
| Total (9) | 3 | 4 | 2 |

SRMAs, systematic reviews and meta-analyses.

**Table S6. CCA Calculation Example**

|  | **A1 *vs.* A2** | **A1 *vs.* A3** | **A2 *vs.* A3** |
| --- | --- | --- | --- |
| Total of included studies (N) | 7 | 5 | 6 |
| Row (r) | 5 | 4 | 5 |
| Columns (c) | 2 | 2 | 2 |
| Calculation result of CCA | 0.4 (40%) | 0.25 (25%) | 0.2 (20%) |

CCA, corrected covered area.
